# Bipolarity in planarians is not induced by space travel

**DOI:** 10.1002/reg2.90

**Published:** 2017-12-05

**Authors:** Ronald Sluys, Giacinta A. Stocchino

**Affiliations:** ^1^ Naturalis Biodiversity Center PO Box 9517 2300 RA Leiden The Netherlands; ^2^ Dipartimento di Scienze della Natura e del Territorio Università di Sassari Via Muroni 25 I‐07100 Sassari Italy

**Keywords:** bipolarity, heteromorphoses, planarians, regeneration, space travel

## Abstract

Available evidence strongly suggests that alternative, Earth‐bound explanations should be sought first for the occurrence of a single bipolar planarian flatworm returning from space travel. Double‐headed worms have been amply documented as arising under experimental conditions as well as spontaneously in stock cultures of planarians.

In this short contribution we show that the claims of Morokuma et al. (2017) that space travel of regenerating pieces of planarian flatworms underlies the induction of bipolar heads and that on Earth the occurrence of such heteromorphoses is extremely rare are unjustified and mostly rest on an eclectic survey of the literature.

From 30 specimens of *Dugesia japonica* Morokuma et al. (2017, Fig. 2A) cut off a head fragment and a tail fragment, thus creating a middle portion of the body containing a major organ of the alimentary system, namely the tubular pharynx. Fifteen of these pharyngeal pieces were sent into space, while the other half were left on Earth as control parts. Upon return from space it turned out that one pharynx piece showed heteromorphic regeneration in that it had grown a new head on either side. Such a bipolar, double‐headed condition was not observed in the control pieces. Morokuma et al. (2017) state that during their extended studies on *D. japonica* they have never encountered a bipolar animal in their colonies or during their experiments. Therefore, they conclude that the bipolar condition must be an effect of space travel and they speculate on the possible underlying mechanism of induction. In subsequent, Earth‐bound experiments on the bipolar worm it turned out that, upon at least two rounds of removing both heads, the pharyngeal piece regenerated the bipolar heads. Morokuma et al. (2017) conclude that this demonstrates a stable, major body‐plan modification, the persistence of which may not be due to space travel but may be a general feature of such heteromorphoses.

A first and obvious comment to be made on the presumed abnormal occurrence of one bipolar worm returning from the space travel experiment concerns the low sample number, i.e., *n* = 1 double‐headed worm. And not only is this part of the report of Morokuma et al. (2017) based on a single event but also on only one experiment with no repeat. Although logistical limitations may have prevented Morokuma et al. (2017) from easily performing repeat experiments, which we fully understand, the situation nevertheless remains that statistical support for the observed phenomenon is lacking. It is true that Morokuma et al. (2017) provide another kind of statistical test, relating to the great number of fragments that they have used in their other, Earth‐bound experiments. But, evidently, that test does not relate to the space travel conditions.

Morokuma et al. (2017) refer to their bipolar space worm as a spontaneously induced and extremely rare phenotype. However, we doubt that there is much spontaneity involved, as the worm was amputated at both ends of its body. It is well known that in freshwater and marine planarians after amputation such segments may give rise to double‐headed worms, whether or not after having been exposed to certain chemicals, physical stimuli or to irradiation (Brønsted, 1969; Chandebois, 1976; Child, 1915; Hauser & Santos, 1985; Morgan, 1902, 1904). This was not only established for species such as *Procerodes lobatus*, *Cercyra hastata*, *Girardia dorotocephala*, *G. tigrina*, and *Cura foremanii*, but also for the model species used by Morokuma et al. (2017), namely *Dugesia japonica* (presumed *Dugesia gonocephala* of Japanese workers such as Teshirogi and Kanatani—see Brønsted (1969) for references and his Figures 42, 43—actually concerns *D. japonica—* see Ichikawa & Kawakatsu, 1964).

Thus, experimentally induced double‐headedness in planarians has been amply documented, including the space‐traveled piece. The experimental procedures followed in these studies may be different. For example, Morokuma et al. (2017) used a large trunk fragment that included the pharynx, whereas in the classical works the induced double‐headedness was generally associated with short fragments which did not include the pharynx and where there was only a small distance between the anterior and posterior facing wounds. However, Morgan (1904) obtained bipolar worms from fragments that included the pharynx and he cites Bardeen, who obtained, in the year before, double‐headed worms more frequently from pharyngeal pieces than from other fragments. Furthermore, Kanatani (1958) obtained bipolar worms, after treatment with demecolcine, from long anterior segments of *D. japonica* (the same model species as used by Morokuma et al., 2017) that included the pharynx, while Levin (2014) described two‐headed phenotypes arising from a large pharyngeal trunk fragment treated with octanol. Further, Hauser and Santos (1985) reported that they had generated Janus heads from short pieces of *Girardia schubarti* that included a portion of the pharynx; it is noteworthy that in these series of experiments many other short fragments of *G. schubarti* that did not include a part of the pharynx did not regenerate bipolar heads.

More recently, a large trunk fragment of *Schmidtea mediterranea*, including the pharynx, regenerated not only a head at the anterior end but also a head at the posterior blastema after inhibition of the protein Smed‐βcatenin‐1 (Petersen & Reddien, 2008). It has been suggested that β*‐*catenin plays a key role in polarity specification in planarians (Gurley, Rink, & Alvarado, 2008; see also Iglesias, Gomez‐Skarmeta, Saló, & Bartscherer, 2008). We doubt that this presumed molecular switch (Gurley et al., 2008) is restricted to blastemas arising at only a few regions along the anterior−posterior axis of the planarian body (see also Morgan, 1904, who reported that heteromorphic heads may appear at any portion of the worm).

In view of these experimental results, as briefly summarized above, we posit that the pharyngeal pieces used by Morokuma et al. (2017) constitute no essential difference with experimental fragments used in other studies.

In contrast to experimentally induced heteromorphosis, spontaneous, natural occurrence of bipolar worms has been observed much more rarely in stock cultures of planarians. However, Jenkins (1963) reported no less than four of such bipolar specimens from a single culture of mature *G. dorotocephala* that had not been subjected to any experimental treatment. One of these worms fissioned and thus gave rise to two animals, each with one head. After 10 days one of these animals again became bipolar; this resembles the presumed stable body‐plan modification reported by Morokuma et al. (2017), as well as Levin (2014 and references therein). That such second or third round amputated fragments regenerated a head at both ends is unsurprising in view of the fact that the pieces contained two pharynges oriented in opposite directions (see Jenkins, 1963; Levin, 2014, and references therein; Morokuma et al., 2017). In our experience, such double‐headed animals with two pharynges behave as two separate, independent individuals.

More recently, the present authors discovered the spontaneous occurrence of two double‐headed planarians in a culture of an unidentified species presumably of the genus *Atrioplanaria* from Sardinia, Italy (see Stocchino et al., 2015; Fig. S1) and one bipolar animal in a culture of a so far unidentified species of *Dugesia* from Liguria, Italy (Fig. 1). With respect to the presumed *Atrioplanaria* it is possible that the particular heteromorphosis it exhibited may concern a rare reproductive strategy (Stocchino et al., 2015).

**Figure 1:**
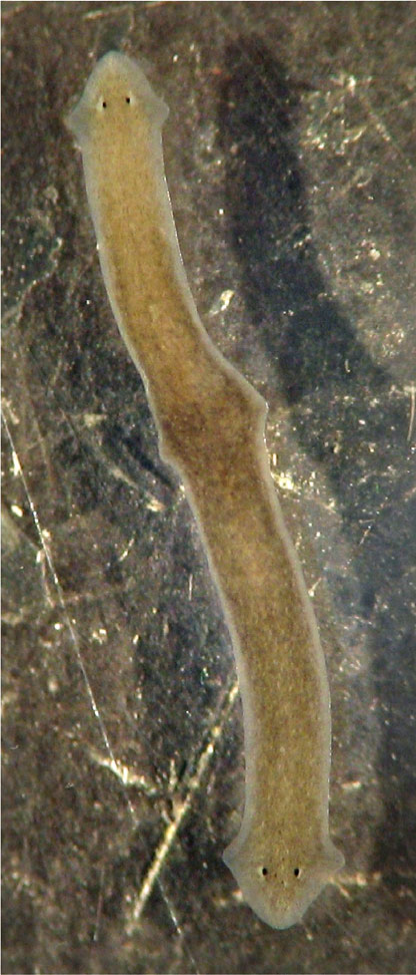
Spontaneously formed bipolar specimen of *Dugesia* sp. Scale bar not available

In their studies on regeneration different workers have used different model species. Therefore, one may be inclined to contemplate the caveat that what applies to one species does not necessarily hold true for another model species. However, although the capability of regeneration is not equally distributed among the triclads, there is yet no indication that the regenerative processes differ among species of planarians. The underlying principle of using model species is that regenerative processes, inducers, releasers, signaling pathways, etc. present in one species occur also in other planarians, until shown otherwise. In fact, planarian species are regularly used as models for genetic discoveries with potential implications even for human development, regeneration, and disease.

One may wish to argue that the spontaneous occurrences of double‐headed worms in planarian cultures are unlikely to be related to the processes described by Morokuma et al. (2017) and that, in addition to Earth‐bound determinants, factors such as microgravity and hypomagnetic environments may play a role. However, in our view the burden of proof for such a point of view or hypothesis rests with the experimenters and as such has not been presented by Morokuma et al. (2017). Until shown otherwise, it is best assumed that the same Earth‐bound processes have effected regeneration of two heads under both experimental as well as more natural conditions.

Interestingly, reversed polarity during reproduction or regeneration occurs also in the flatworm‐like metazoans *Convolutriba retrogemma* and *C. macropyga*. In these species asexual reproduction involves a budding process in which buds are generated with a body axis orientation that is the reverse of that of the parent animal (Sikes & Bely, 2010).

Thus, studies on the culturing and regeneration of planarians show that the purportedly extremely rare occurrence of a single bipolar worm returning from space travel is simply an example of a more common, Earth‐bound phenomenon. Therefore, causal explanations for this kind of heteromorphosis should be sought first in mechanisms and factors that are unrelated to a reduced geomagnetic field or microgravity. That space travel has little to do with the growth of heteromorphic heads was already foreshadowed by the fact that exposure of the pharyngeal pieces of the worms to conditions in the International Space Station started only approximately 78 h after amputation (Morokuma et al., 2017) and that thus regenerative processes were already well in progress.

In conclusion, the available collection of data on regeneration in triclads clearly indicates that (1) the evidence for the effect of microgravity presented by Morokuma et al. (2017) is very weak and (2) the study failed to take sufficiently into account similar results documented in the literature. In addition, we believe that prudent application of the principle of parsimony in science prescribes that for causal explanations we should look first for Earth‐bound processes and determinants, especially given the 78 h delay and our knowledge that the phenomenon of bipolarity occurs with some regularity on Earth. Such Earth‐bound process hypotheses do not require additional ad hoc hypotheses, such as microgravity, and are able to explain a wider range of phenomena, i.e., those occurring on Earth as well as the single double‐headed animal returning from space travel.

## CONFLICT OF INTEREST

The authors declare that they have no conflicts of interest

## Supporting information


**Figure S1** Spontaneously formed bipolar specimen of a presumed *Atrioplanaria* sp. Scale bar not available
