# Supplementary figures and images for: Bipolarity in planarians is not induced by space travel
Source: Regeneration (Oxf). 2017 Dec 5;4(4):153–5. doi: 10.1002/reg2.90 (PMC5743782; doi:10.1002/reg2.90)

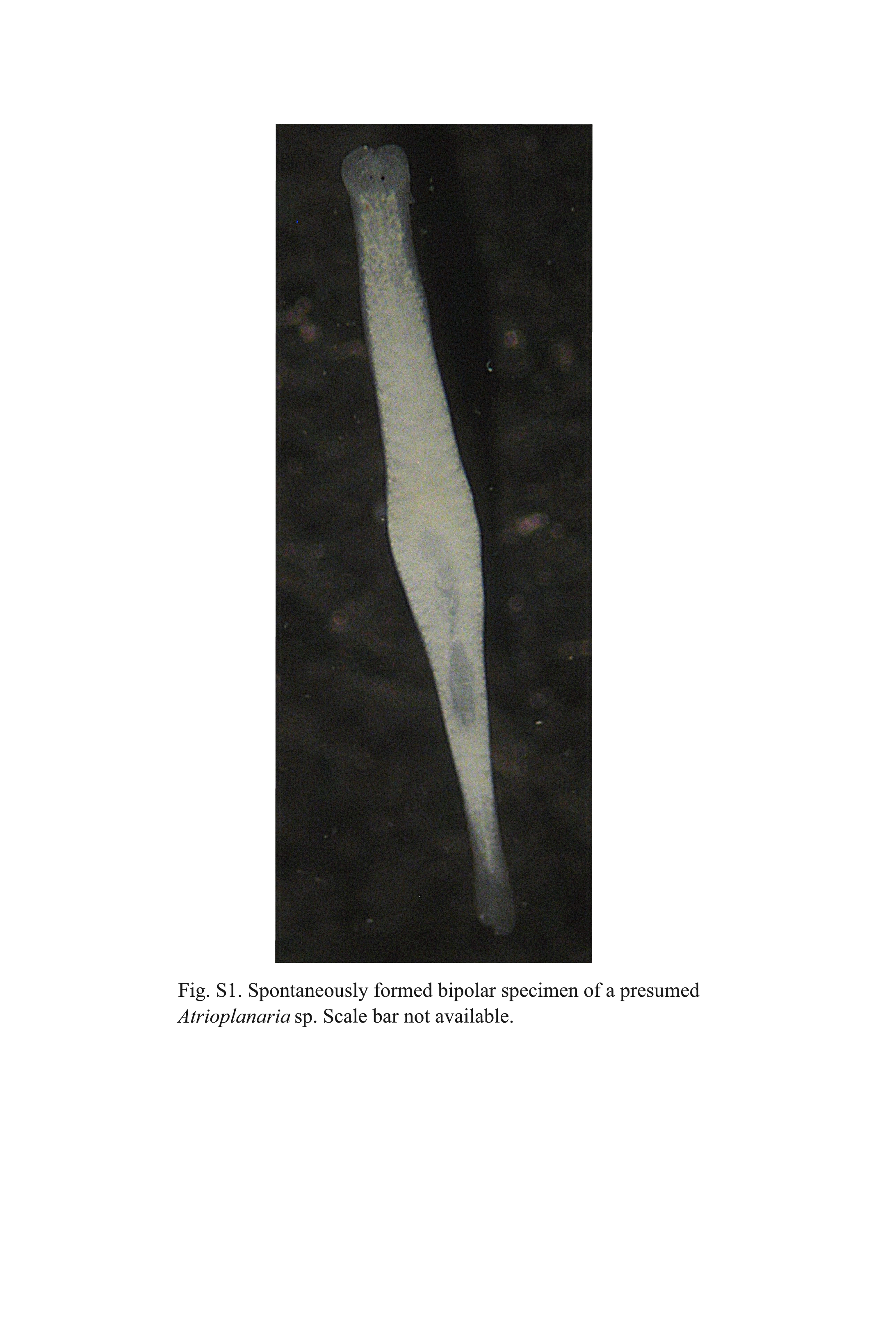

Supplement: Supplementary file 1 — Figure S1 Spontaneously formed bipolar specimen of a presumed Atrioplanaria sp. Scale bar not available [file REG2-4-153-s001.tif]
